# Have lessons from past failures brought us closer to the success of immunotherapy in metastatic pancreatic cancer?

**DOI:** 10.1080/2162402X.2015.1112942

**Published:** 2015-12-18

**Authors:** Laura Rosa Brunet, Thorsten Hagemann, Andrew Gaya, Satvinder Mudan, Aurelien Marabelle

**Affiliations:** a Immodulon Therapeutics Ltd, Stockley Park, Uxbridge, UK; b Deparment of Clinical Oncology, Guy's and St Thomas' NHS Foundation Trust, London, UK; c Royal Marsden Hospital, Fulham Road, London, UK; d INSERM, U1015, Villejuif, France; e Center of Clinical Investigations in Biotherapies of Cancer (CICBT) 507, Villejuif, France; f Drug Development Department, Gustave Roussy Cancer Campus, Villejuif, France

**Keywords:** Adaptive immunity; immunotherapy micro-organism; innate immunity; pancreatic cancer

## Abstract

Pancreatic cancer is extremely resistant to chemo- and radiation-therapies due to its inherent genetic instability, the local immunosuppressive microenvironment and the remarkable desmoplastic stromal changes which characterize this cancer. Therefore, there is an urgent need for improvement on standard current therapeutic options. Immunotherapies aimed at harnessing endogenous antitumor immunity have shown promise in multiple tumor types. In this review, we give an overview of new immune-related therapeutic strategies currently being tested in clinical trials in pancreatic cancer. We propose that immunotherapeutic strategies in combination with current therapies may offer new hopes in this most deadly disease.

## Introduction

Pancreatic cancer is characterized by an extremely poor survival rate, with up to 80% patients dying within the first year of diagnosis. In contrast to the positive outlook reported in other cancers in recent years, survival prospects have remained bleak and in Europe the trend is stubbornly negative in both sexes.^1^ Indeed, the 5-y mortality rate for pancreatic cancer is over 95%.^2^ Surgical resection is the only potentially curative treatment. However, overall survival (OS) rate even after surgery remains poor, as over 75% of patients with localized disease, amenable to complete surgical resection, die of local or metastatic recurrence within 5 y^3,4^ Unfortunately, because pancreatic cancer is usually diagnosed at an advanced stage due to lack of specific symptomatology early in the disease, surgery is suitable for only a minority of patients. At the time of diagnosis, only 15–20% of patients present with operable disease whereas about 40% are found to have locally advanced, unresectable disease and approximately 45% have metastatic disease.^5^

For patients unable to resort to surgery, these tumors represent particularly difficult therapeutic challenges, as they tend to be resistant to current chemo- and radiation-therapy strategies. This feature is due to the inherent genetic instability of pancreatic cancer cells, the immunosuppressive microenvironment at the tumor site and a remarkable desmoplastic reaction characterizing this cancer and renders it impenetrable to most chemotherapies. Ultimately, it is the development and spread of metastases, which leads to patients' death. Hence, controlling metastatic spread or increasing susceptibility of metastases to treatment may become an increasingly attractive avenue of research to improve survival.

### Treatment options—chemotherapy

Gemcitabine monotherapy has been the standard treatment for pancreatic cancer since the late nineties. In a small pivotal clinical trial of 126 patients, treatment with gemcitabine, a nucleoside analog, was compared to bolus administration of 5-fluorouracil. Gemcitabine mediated a significant, albeit modest, effect (5.6 vs. 4.4 mo; *p* = 0.0025) on OS as well as improvements on quality of life, performance status and pain control.^6^ The primary efficacy measure was clinical benefit response, which was a composite of measurements of pain (analgesic consumption and pain intensity), Karnofsky performance status and weight. Clinical benefit required a sustained ( ≥4 weeks) improvement in at least one parameter without worsening in any others.

Over the past two decades, gemcitabine has been the backbone for the addition of many compounds. Combination doublets of gemcitabine with other chemotherapeutic agents (capecitabine, irinotecan, oxaliplatin and cisplatin) have shown limited clinical effects over gemcitabine monotherapy.^7^ A recent meta-analysis of 26 studies (with a total of over 8800 patients), reported a significantly lower objective response rate (ORR) (Relative Risk (RR) 0.72; 95% CI 0.63–0.83; *p* < 0 .001), and lower 1-y OS (RR 0.90; 95% CI 0.82–0.99; *p* = 0.04) of monotherapy compared only to doublet treatment with fluoropyrimidine, but at the cost of increased toxicity.^8^ The addition of angiogenic inhibitors (bevacizumab, axitinib and aflibercept) has also failed to demonstrate any significant OS benefit.^9-11^ The combination with erlotinib (Tarceva), a tyrosine kinase epidermal growth factor receptor inhibitor, has shown a very small clinical benefit in OS (6.4 vs. 6 mo; *p* = 0.028) and progression-free survival (PFS) (3.8 vs. 3.5 mo; *p* = 0.006) but at the expense of significant skin and gastrointestinal (GI) toxicities and considerable cost.^12^ For these reasons, gemcitabine still is for most patients, especially those with poor performance status, the preferred and only treatment option.

In 2010, a randomized Phase III study, Prodige 4-ACCORD 11, reported on 336 untreated metastatic pancreatic ductal adenocarcinoma (PDAC) patients with good performance status (ECOG score of 0 or 1, normal bilirubin, good bone marrow and renal function) treated with FOLFIRINOX compared to gemcitabine alone.^13^ Patients receiving the combination treatment had significantly longer OS (11.1 vs. 6.8 mo; Hazard ratio (HR)=0.57; 95% CI 0.45–0.73; *p* < 0 .001). Moreover, ORR (31.6% vs. 9.4%; *p* < 0 .001) and PFS were also significantly improved (6.4 vs. 3.3 mo; HR 0.47; 95% CI 0.37–0.59; *p* < 0 .001). Unfortunately, these improvements were countered by a raised incidence of various grade 3–4 toxicities, including febrile neutropenia (5.4% vs. 0.6%; *p* = 0.009), thrombocytopenia (9.1 vs. 2.4; *p* = 0.008), peripheral neuropathy (9% vs. 0%; *p* = 0.001), vomiting (14.5% vs. 4.7%; *p* = 0.002), diarrhea (12.7 vs. 1.2; *p* = 0.0001), thromboembolic events (6.6% vs. 4.1%).

The most recently FDA-approved treatment option for patients with advanced stage pancreatic cancer is Abraxane, albumin-bound paclitaxel (nab-paclitaxel) in combination with gemcitabine. A Phase III study (MPACT) reported in 2013 on the effect of gemcitabine plus nab-paclitaxel versus gemcitabine alone, in 861 untreated metastatic PDAC patients.^3^ OS was significantly improved (8.5 vs. 6.7 mo; HR=0.72; 95% CI 0.62–0.83; *p* < 0 .001) as well as one-year survival rate (35% vs. 22.2%), PFS (5.5 vs. 3.7; HR=0.69; 95% CI 0.58–0.82; *p* < 0 .001) and ORR (23% vs. 7%; *p* < 0 .001). These significant improvements did not increase treatment-related deaths, which were similar in both groups (4% for each) but grade 3–4 neutropenia (38% vs. 20%), fatigue (17% vs. 7%), neuropathy (17% vs. <1 %) were all higher in the combination group. It is noteworthy to mention that in subgroup analyses of patients with poorer performance status (Karnofsky performance score of 70 and 80) and more bulky disease (liver metastases, >3 metastatic sites), the benefit afforded by this combination was greater.

These clinical developments of the last few years have provided added options for treatment of metastatic pancreatic cancer. However, any survival improvements have come at the expense of toxicity, which are somewhat limiting the general applicability of these therapies due to their effect on patients' performance score and added treatment costs due to toxicities to the health services. For these reasons, there is the urgent need for further therapeutic strategies to improve on patients' survival as well as quality of life. Additionally, the benefits of combining chemotherapy and, or radiotherapy with immune modulators to enhance response in patients has not yet been fully understood. Further investigation may provide much needed insight on effective therapeutic combinations and treatment schedules.

### Treatment options—immunotherapy

Since the end of the 19th century, many attempts have been made to harness immunity in the battle against cancer. Following on from the early work of European physicians observing a correlation between severe inflammatory responses and cancer regression, William Coley was the first to systematically utilize this association in cancer therapy, by treating his patients with Coley Toxins, a preparation of killed *Streptococcus pneumoniae* and *Serratia marcescens*.^14^ More recently, clinical responses have been well documented in some malignancies with Bacillus Calmette–Guerin (BCG), a strain of *Mycobacterium bovis*, currently approved for use in non-invasive transitional cell carcinoma of the bladder.^15^ While medical oncologists have been skeptical of immunotherapy for some time due to the many negative results in solid malignancies, hematologists have successfully harnessed the power of the immune system to induce complete, long-term remissions in patients with leukemia.^16^ Beyond the therapeutic failures, part of the immunotherapy skepticism in the oncology community was explained by the confusing diversity of strategies tested. Indeed, cancer immunotherapy strategies have included active (e.g., vaccines) and passive (e.g., monoclonal antibodies) immunotherapies which could be either specific (e.g., adoptive T cell) or non-specific (e.g., cytokines) for the cancer treated, the allogeneic transplantation of immunity (e.g., bone marrow, haplo-identical NKs) being a mix of all these strategies. To date, the use of immunotherapy in pancreatic cancer has been rather disappointing. However, recent advances in our understanding of molecular immunology and the interplay between the immune system and cancer have led to some exciting and promising developments. Here, we will review several different immunotherapy strategies used. Due to space limitations, passive immunotherapies are beyond the scope of this article.

### Immune responses in pancreatic cancer

The clinical and pre-clinical data suggesting a major role for immunity in pancreatic cancer are now compelling. Pancreatic cancer patients are able to generate both B and T cells recognizing antigens (Ag) expressed on autologous pancreatic tumor cells.^17-20^ These include Wilms' tumor gene 1 (WT1) (75% of patients),^21^ mucin 1 (MUC1) (over 85% of patients),^22^ human telomerase reverse transcriptase (hTERT) (88% of patients),^23^ mutated K-RAS (73% of patients),^24^ and carcinoembryonic antigen (CEA) (over 90% of patients).^25^ Furthermore, sera from patients contain antibodies to tumor associated Ags, MUC-1 and mesothelin, in particular.^26,27^ Interestingly, pre-invasive pancreatic lesions are characterized by infiltration of immune suppressor cells and absence of immune effector cells, suggesting that tumor immunity may be defective already from the inception of pancreatic cancer development.^28^ The notion that defective immunological responses are responsible for cancer development is supported by evidence from animal models, which confirms the existence of immune surveillance mechanisms mediating responses which suppress cancer. For example, mice lacking interferon (IFN) ^29^ and perforin,^30^ vital components for cytotoxic activity, are prone to develop cancer. Moreover, the adaptive immune system can recognize and eliminate malignant cells; in experimental models, it can limit growth of spontaneous and transplanted tumors.^31^ Protective Ag-specific T cells can also be detected in human cancers.^32^ However, their effects can be inhibited by the tumor microenvironment. In pancreatic cancer, tolerance to tumor Ag may occur due to Ag persistence, downregulation of major histocompatibility antigens (MHC) which prevents effective Ag presentation or increased infiltration of cells with immunosuppressive properties such as Ag-specific regulatory T (Treg) cells, tumor-associated macrophages (TAMs), myeloid-derived suppressor cells (MDSCs) and tumor-associated fibroblasts.^33,34^ The accumulation of MDSCs and Tregs, as well as the alterations to checkpoint pathways which control immune responses development, have been shown to be closely related to the extent of disease, to correlate with disease stage and to predict survival.^35-38^ Non-specific ‘innate’ tolerance can also be maintained by innate immune cells through the production of anti-inflammatory and immunosuppressive mediators and downregulation of Ag-presenting cell activity.^39^

### Inhibitors or agonists of checkpoint control

To target solid malignancies effectively, tumor-specific T cells must avoid negative regulatory signals that inhibit their activation or induce tolerance in the form of anergy or exhaustion. Cytotoxic T lymphocyte associated protein-4 (CTLA-4) and programmed cell death protein-1 (PD1) are major negative co-stimulatory molecules expressed on activated T cells.^40^ Antibodies targeting these suppressive co-stimulatory receptors block inhibitory signals and prolong the life of activated T cells as well as induce T cell proliferation. The discovery of immune checkpoint blockade inhibitors is an exciting advance in the field of immunology that has pushed the clinical landscape to make significant progress in cancer immunotherapy.

Following on the clinical success of treatment with an anti-CTLA-4 inhibitor, ipilimumab, in melanoma,^41^ this strategy was tested in Phase II clinical trials in advanced pancreatic cancer using ipilimumab (NCT01473940), pidilizumab (anti-PD1 mAb) (NCT01313416) and CP-870,893 (a selective agonist mAb of the CD40 receptor) (NCT00711191). Whereas results with ipilimumab have suggested no direct radiological tumor responses,^42^ treatment with CP-870,893 in combination with gemcitabine led to activation of the immune system and tumor response in a small cohort of patients with unresectable pancreatic cancer.^38^ Four patients out of 22 chemo-naive pancreatic cancer patients achieved a partial response, while 6 patients showed a PET response with more than 25% decrease in fluorodeoxyglucose uptake within the primary pancreatic tumor. However, responses observed in metastatic lesions were heterogeneous.^43^ Several trials are now recruiting to investigate the combination of two checkpoint blockade inhibitors (CTLA-4 and PD1/PDL1 blockade) or combination with small molecule inhibitors to overcome the immunosuppressive tumor microenvironment. An ongoing study (NCT02301130) currently investigates the combination of mogamulizumab (an anti-CCR4 mAb) with either MEDI4736 (anti-B7H1 mAb) or tremelimumab (anti-CTLA-4 mAb) to overcome the immunosuppression in pancreatic cancer. This is a constantly evolving clinical research area aiming to find feasible combinations to restore and increase the activation of adaptive and innate immunity.

To date, it is still not understood why certain solid malignancies demonstrate a better clinical response to checkpoint blockade inhibitors than others. This appears to be particularly true for malignancies of the GI tract where anti-PD-1 mAb has activity in the esophageal and gastric cancers, but no activity in the colon (except those carrying microsatellite instabilities) and pancreatic cancers. More combination treatments need to be clinically investigated in this area to provide patients with suitable alternative options.

### T cell therapies

Recently, cancer immunotherapy has focused on the activation of adaptive immunity. MUC-1-specific autologous T cells, isolated from patient peripheral blood mononuclear cells (PBMCs), were expanded by incubation with a MUC-1-presenting cell line prior to administration to pancreatic cancer patients. The mean survival time for unresectable patients in this study was 5 mo.^44^ In a similar study, PBMC-derived mature DCs from a pancreatic cancer patient were pulsed with MUC-1 peptide. The pulsed DCs were administered in combination with MUC-1-specific T cells to patients with unresectable or recurrent pancreatic cancer. A complete response was observed in one patient with lung metastases and the mean survival time of the whole group was 9.8 mo, suggesting that the addition of pulsed DCs may have beneficial effects.^45^

### Chimeric antigen receptors

An alternative to Ag-specific expansion is the lentiviral transduction of patient T cells with a chimeric antigen receptor (CAR) specific for a tumor Ag. CARs are transmembrane proteins comprising an antibody-derived single-chain variable fragment (scFv) specific for a tumor Ag fused to a hinge region, a spacer, a membrane spanning element and signaling domain.^46^ Often, the intracellular signaling domain contains the signaling motifs from multiple costimulatory molecules (41BB, OX40, CD28). This allows for both T cell receptor and costimulatory signaling cascades to be initiated, leading to optimal T cell activation. The resulting T cells recognize the tumor Ag in its native form and do not rely on its presentation by MHC class I, which is often downregulated in cancer. Several CARs have been created with specificity for mesothelin, CEA, MUC-1 and Her-2/neu and Phase I/II trials (NCT01583686, NCT02465983, NCT02349724) are ongoing.^47-49^ Although this process results in a large population of Ag-specific T cells, adoptive immunotherapy is currently an expensive and time-consuming process in comparison with tumor Ag-based vaccine, systemic immune stimulation or monoclonal antibody therapies.

### Vaccination therapies

Although Ag-specific immune responses can be detected in cancer patients undergoing tumor cell vaccination therapies, this approach has not delivered great successes in pancreatic cancer with the notable exception of post-surgical vaccination therapy which has shown a beneficial impact in pancreatic cancer patients with absent or minimal residual disease.^50^ Many studies using whole-cell, DNA as well as peptide vaccines have been performed or are ongoing, but to date, there is no vaccine therapy showing benefit in metastatic pancreatic cancer. Most recently, a large Phase III trial (TELOVAC) comparing gemcitabine and capecitabine with or without telomerase peptide vaccine GV1001 in patients with locally advanced or metastatic pancreatic cancer failed to report any clinical benefit.^51^ Although the TeloVac trial did not demonstrate any significant difference in OS and PFS between treatment arms, it demonstrated that the vaccine could prevent patient deterioration. Interestingly, further analysis of potential immunological biomarkers did demonstrate that baseline eotaxin levels predicted median OS in the concurrent gemcitabine and Capecitabine with GV1001 group. Whereas high eotaxin levels at baseline correlated with a longer OS in this study, sequential chemo-immunotherapy did not show any correlation between eotaxin levels and OS. A better understanding of the right immunological response profile would be advantageous for future clinical trial so recruitment of potential responders can be better guided.

For this reason, we will not expand further on vaccination therapies, aside from mentioning the case of a 77-y-old patient who was treated with survivin-based peptide vaccination and had a partial response in liver metastases at 6 mo and complete response at 8 mo.^52^ However, the patient developed recurrent disease after being weaned off the vaccine therapy.

### Utilizing bacteria for cancer therapy

The idea of harnessing immunity by inducing repeatedly infectious stimuli was born over 100 y ago. This concept was recently revisited with the suggestion that repeat exposures to microbes, which may induce non-specific acute inflammation and febrile episodes activate immune memory of antigenic changes important for cancer immunosurveillance.^53^ For example, infections and acute inflammations generate abnormal Ags that activated DC carry to the draining lymph nodes, where they stimulate adaptive immunity and immune memory. This immune memory can be reactivated by tumor Ags and depending on the strength of the memory and of the reactivation, cancer cells may be either eliminated or kept in equilibrium. Two forms of bacterial formulations have been used in the clinic and need to be mentioned here.

### Listeria monocytogenes

To date, this bacterium has been used either as a vehicle to deliver a specific epitope (mesothelin) via expression in live-attenuated *Listeria monocytogenes*.^54^ or as a radioactive-labeled formulation.^55^ While the latter has only shown efficacy in pre-clinical models, the live-attenuated *L. monocytogenes*-expressing mesothelin (CRS-207) in combination with low-dose cyclophosphamide and GVAX pancreas (granulocyte-macrophage colony-stimulating factor-secreting allogeneic pancreatic tumor cells) was compared to cyclophosphamide plus GVAX only, in 90 patients with metastatic pancreatic cancer. In the per-protocol analysis of patients who received at least three doses (two doses of cyclophsphamide/GVAX plus one of CRS-207 or three of cyclophsphamide/GVAX), OS was 9.7 vs. 4.6 mo (*p* = 0.02). Enhanced mesothelin-specific CD8^+^ T cell responses were associated with longer OS in both groups.^54^ This treatment was given ‘breakthrough therapy’ designation by the FDA in 2014. Two clinical trials are planned, one investigating the addition of anti-PD-1 antibodies to the combination (NCT02243371), the other the addition of GVAX plus anti-CTLA-4 antibody in patients receiving FOLFIRINOX (NCT01896869).

In addition to the immune-related therapeutic strategies outlines in this review, we would like to mention the randomized, controlled STELLAR trial (Safety and Therapeutic Efficacy of Live-attenuated Listeria/GVAX with anti-PD-1 Regimen) which has enrolled approximately 88 patients with metastatic pancreatic cancer who have been treated previously with one line of chemotherapy. CRS-207/GVAX Pancreas vaccine and nivolumab treatment is compared to CRS-207/GVAX Pancreas vaccine alone in this Phase II trial for impact on OS. Secondary endpoints include evaluation of clinical and immune response and safety (NCT02243371).

Lastly, even though not immune-related the clinical development of evofosfamide both as a monotherapy and in combination with chemotherapy treatments needs mention. Evofosfamide is currently under evaluation in a Phase III trial (MAESTRO) in combination with gemcitabine versus gemcitabine and placebo in patients with locally advanced unresectable or metastatic pancreatic cancer. This Phase III trial is being conducted under Special Protocol Assessment (SPA) agreements with the FDA. Based on current projections, the number of protocol-specified events may be reached in the second half of 2015, with the results of the primary efficacy analyses available shortly thereafter. The FDA and the European Commission have granted evofosfamide Orphan Drug Designation for the treatment of pancreatic cancer.

### Mycobacterium obuense

As above mentioned, mycobacteria have demonstrated antitumor activity in both pre-clinical and clinical settings and intra-vesical *Mycobacterium bovis* in the form of BCG is standard of care for non-muscle-invasive Bladder Cancers. *Mycobacterium obuense* another member of the *Mycobacterium* genus, is a non-pathogenic saprophytic mycobacteria. IMM-101 is derived from heat-killed *Mycobacterium obuense* (NCTC 13365), which can induce both innate and adaptive immune responses to boost antitumor immunity. Unlike *L. monocytogenes*, IMM-101 is not used as a vehicle to deliver either radioactive or tumor-specific Ags. Rather, IMM-101 contains a wide variety of pathogen-associated molecular patterns (PAMPs), including proteins, lipoproteins and carbohydrate Ags which may ensure activation of a wide pool of memory CD8^+^ cells, which while re-circulating may then amplify the immune response and target metastases and tumor Ags.

In preclinical models, treatment with IMM-101 appeared to increase the secretion of IFNγ and cytotoxic mediators such as perforin and granzyme B at the tumor site, the draining lymph nodes and in the spleen in tumor-bearing mice.^56^ Most importantly, these modulatory effects are induced when the preparation is administered subcutaneous away from the tumor site. In a recent randomized, open-label, proof-of-concept, Phase II trial in advanced pancreatic cancer (IMAGE 1), the combination of IMM-101 with gemcitabine was tested (NCT01303172).^56^ Patients with advanced pancreatic cancer and a WHO score of 0–2 were assigned randomly in a 2:1 ratio to receive IMM-101 plus gemcitabine or gemcitabine alone. A total of 110 patients were randomized, 75 to receive IMM-101 plus gemcitabine and 35 gemcitabine alone. In the pre-defined sub-group of patients with metastatic disease (n = 92), median OS was increased significantly by 59% to 7 mo in the IMM-101 plus gemcitabine group (n = 64) compared to 4.4 mo in the gemcitabine alone group (n = 28) (HR 0.54; 95% CI 0.33–0.87; *p* = 0.01). A highly significant 91% increase in median PFS from 2.3 mo in the gemcitabine group to 4.4 mo in the IMM-101 plus gemcitabine group was observed (HR 0.46; 95% CI 0.28–0.75; *p* = 0.001). Patients with locally advanced disease at the time of enrolment were eligible for the study and 18 such patients were included, 11 randomized to IMM-101 plus gemcitabine and 7 to gemcitabine alone. This sub-group was too small to draw firm conclusions but there was no evidence for a beneficial effect of IMM-101, which is consistent with preliminary findings from preclinical studies that indicated a more profound effect of IMM-101 on metastases than on the primary tumor.^56^

### The current challenge of immunotherapy in pancreatic cancer: finding synergistic combination therapies

Treatment of advanced pancreatic cancer has concentrated on single agent therapies or combination of compounds within the same class (e.g., cytotoxics). Perhaps, a polyvalent approach would be more appropriate for such a heterogeneous disease.^57^ Chemotherapies exert various effects on the immune system that could be exploited to enhance the efficacy of immunotherapies. It had long been assumed that immune-stimulatory compounds could not be used in combination with immunosuppressive chemotherapies, but recent evidence has challenged this dogma. Chemotherapies could be used to condition the immune system and the tumor milieu to create an environment where immunotherapies have a better chance of success.

Three ways are proposed by which chemotherapy could promote immune responses and act in synergy with immunotherapies^58^: (a) deplete immune suppressive cells; (b) increase tumor immunogenicity by releasing tumor Ags due to cytotoxic activity; (c) direct activation of T cells. Any one of these effects could enhance the tumor-specific immune response elicited by immunotherapeutic agents, and some chemotherapies may even work through multiple mechanisms. A recent publication by García-Martínez and colleagues,^59^ demonstrated that in a group of 121 neo-adjuvantly treated breast cancer patients, characterization of the immune cell subpopulation profiles by immunohistochemistry-based computerized analysis, identified groups of patients characterized by high response (in the pre-treatment setting) and poor prognosis (in the post-treatment setting). Similarly, immunologic factors were highly significant predictors of therapy response in the GeparSixto trial in breast cancer patients treated with carboplatin.^60^

Cyclophosphamide has been described to affect subsets of CD4^+^ T cells.^61^ In 2005, it was revealed that this drug efficiently depletes CD4^+^CD25^+^Treg cells at low doses ^62^ while leaving CD4^+^CD25^−^ and CD8^+^ T cells unaffected. Gemcitabine has been proposed to deplete MDSCs. Both drugs have been reported to induce only a transient depletion of the respective cell populations.^63^ Interestingly, since MHC class II positive MDSCs can promote Treg activation, gemcitabine could potentially reduce multiple suppressor cell types. Vincent et al. ^63^ examined gemcitabine, cyclophosphamide, 5-fluorouracil and paclitaxel among others in pre-clinical models of cancer. 5-fluorouracil induced apoptosis of GR^+^CD11b^+^ MDSC and was more potent than gemcitabine. 5-fluorouracil and gemcitabine have also been reported to increase immunological visibility of tumors by increasing expression of tumor Ags.^64^ Recently, low-dose paclitaxel has been shown to have stimulatory effects on the immune system.^65^ In murine models, paclitaxel is a ligand for TLR4 on DCs, mediating a direct effect on the immune system.^66^ In addition, paclitaxel has also been shown to enhance activation of human DCs independent of TLR4 receptor engagement.^67^ These findings supporting synergy between effects mediated by chemotherapy and the immune system, raise the question as to whether the success of FOLFIRINOX is potentially related to the combination of chemotherapy with additional, although not intentionally given for this purpose, immune modulation via G-CSF, which is administered to prevent the neutropenia caused by FOLFIRINOX.^13^ Similarly, Abraxane might promote some of its efficacy through the immune modulatory effects of paclitaxel. These observations may lead the way for further investigating the right combination of immunotherapies and chemotherapy. The good results presented at ASCO 2015 of anti-PD-1 and anti-PD-L1 combinations with chemotherapy in non-small cell lung cancer (NSCLC) support this idea: such combinations had an ORR above 60%, that is higher than the sum of the ORR obtained with either therapy separately (ORR of around 30% for chemotherapy alone, and 20% for anti-PD-1/PD-L1 mAb).^68,69^

### In situ immunization: back to the future

One striking observation that can be made when looking at the clinical development of immunotherapies is the fact that although they rely on disruptive mechanisms of action, people keep administering them like conventional therapies (e.g., anti-CTLA-4 or anti-PD-1 administered intra-venously (IV) every 3 weeks). Therefore, it is not surprising that systemic activation of the immune system (e.g., sub-cutaneous IL-2 or ipilimumab+nivolumab) generate a very high level of drug related grade 3–4 off-target toxicities. Intra-tumoral injections of immunostimulatory products could be a good way to use a tumor as its own vaccine and to locally prime an antitumor immune response which could subsequently act on distant, non-treated tumor sites.^70^ This *in situ* immunization (also called *in situ* vaccination) strategy is likely what William Coley was doing at the end of 19th century, and long-term tumor responses were reported in multiple, injectable, cancer types.^71,72^ Besides, bladder cancer use, intra-tumoral BCG has also demonstrated significant antitumor activity in melanoma and squamous cell carcinoma of the head and neck.^73-75^ A small clinical report of intra-peritoneal BCG also reported activity in pancreatic cancers.^76^ Interestingly, the study of the effects of mycobacteria in murine tumor models led to the identification of Toll-like receptor 9 (TLR-9) agonists (CpG-rich DNA motifs).^77,78^ Recently, the combination of intra-tumoral CpG combined with low dose irradiation (2 × 2 Gy) has demonstrated antitumor activity in patients with B cell and T cell lymphoma, including activity at distant, non-treated, tumor sites.^79,80^ Also, clinical reports have shown that radiotherapy could overcome anti-CTLA-4 resistance in patients with metastatic melanoma, inducing tumor responses in both irradiated and non-irradiated lesions, through the so-called ‘abscopal effect.’^81,82^ More recently, the combination of local radiotherapy with systemic (s.c.) GM-CSF has been shown to generate abscopal activity in NSCLC, thymic and breast cancers.^83^ Radiotherapy could indeed have a more important role to play in the immunotherapy of metastatic pancreatic cancer. Currently, its role (low dose per fraction, 6 weeks treatment) is for local consolidation following chemotherapy for borderline resectable or locally advanced tumors, with the sole intention of causing cell death by oxidative effects and DNA double strand breaks. Ionizing radiations have the ability to convert the irradiated tumor into an ‘immunogenic hub’—acting in effect like an autologous tumor ‘vaccine.’^84^ At higher doses per fraction, the radiobiology changes due to alternative modes of action.^84,85^ After radiation exposure, tumor cell death includes apoptosis and necrosis as well as autophagy and mitotic catastrophe. Importantly, radiation has been shown to induce an immunogenic cell death, characterized by three molecular signals that promote uptake of dying cells by DCs, cross-presentation of the tumor-derived Ags to T cells and activation of antitumor T cells, exposure of calreticulin on the tumor cell surface, release of high-mobility group protein B1 (HMGB1), and release of ATP.^86^ Furthermore among genes that are upregulated post-radiation are those controlling expression of growth factors, cytokines, chemokines, and cell surface receptors that modulate the interaction of the tumor with the immune system.^87,88^ Thus, radiation may have important systemic effects beyond its local actions.

In addition to excellent local control of disease, high-dose per fraction radiotherapy—stereotactic body radiotherapy (SBRT)—also appears to impact disease outside the irradiated volume. This is likely to be an example of the abscopal effect, resulting from the stimulation of T cell immunity by tumor Ags released by SBRT, leading to the eradication of occult regional micro-metastases. Significant induction of low-density lipoprotein (LDL)-enriched ceramide, secretory sphingomyelinase (S-SMase), tumor necrosis factor-related apoptosis-inducing ligand (TRAIL), and TNF-α in serum from patients treated with SBRT, suggests these bystander effects may have a role in overall tumor response. In view of these encouraging results, the combination of SBRT and immunotherapy in humans is currently being investigated in several studies and should include pancreatic cancer. However, the identification of the right irradiation dose and regimen for optimal immune activation remains unclear and pre-clinical models have brought contradictory results so far.^89^

Only 20–30% of patients generate objective responses in many cancer types with anti-PD-1/PD-L1 therapy and no activity has been reported so far in pancreatic cancers. Therefore, the current challenge in cancer immunotherapy is to overcome primary resistance to immune checkpoint blockade therapy. One way could be to increase the intra-tumoral concentration of these immunostimulatory monoclonal antibodies. This could be a good way to increase T cell activation *in situ* while preventing systemic exposure and off-target toxicity. Interestingly, a recent report at ASCO 2015 has shown strong activity of *in situ* ipilimumab with IL-2 with abscopal effect seen in 75% of patients with metastatic melanoma.^90^ It becomes clear now that the *in vivo* activity of immune checkpoint targeted monoclonal antibodies rely on the presence of FcγR positive cells within the tumor micro-environment (which are mostly myeloid cells, notably macrophages) (see ^91-92^ for review). A good way to switch myeloid cells from a tolerogenic phenotype to an activated Ag-presenting cell phenotype (MHC class I & II high, upregulation of CD80/86) is to stimulate them with PAMPs. Therefore, it would make sense to combine intra-tumoral injections of PAMPs with immune checkpoint targeted antibodies. Indeed, several pre-clinical results have demonstrated the ability of either TLR agonists or oncolytic virus (providers of PAMPs) to overcome immune checkpoint blockade resistance.^93,94^ This strategy is currently tested in several ongoing clinical trials (Table 1) and should be specifically developed in patients with pancreatic cancers where the stroma modification seems critical for efficient immunotherapy.

**Table 1.** 

*In situ* source of PAMPs	Immunostimulatory partner	Cancer types	Trial
T-vec *(Herpes simplex virus type 1 oncolytic virus, Amgen)*	Anti-CTLA-4 (ipilimumab, BMS)	Melanoma	NCT01740297
	Anti-PD-1 (pembrolizumab, Merck)	Melanoma	NCT02263508
	radiation	STS	NCT02453191
DNX-2401 *(Oncolytic adenovirus, DNAtrix)*	IFNγ	Glioblastoma	NCT02197169
HF10 *(HSV1 oncolytic virus, Takara)*	Anti-CTLA-4 (ipilimumab, BMS)	Melanoma	NCT02272855
CAVATAK *(Oncolytic Coxsackievirus A21, Viralytics)*	Anti-CTLA-4 (ipilimumab, BMS)	Melanoma	NCT02307149
BCG *Mycobacterium bovis*	Anti-CTLA-4 (ipilimumab, BMS)	Melanoma	NCT01838200
Poly-ICLC *(TLR7–8 agonist, Oncovir)*	Flt3-Ligand (CDX-301, Celldex)	B cell Lymphoma	NCT01976585
SD-101 *(TLR9 agonist , Dynavax)*	Anti-CTLA-4 (ipilimumab, BMS)	B cell Lymphoma	NCT02254772
	radiation	B cell Lymphoma	NCT02266147

## Closing remarks

Therapeutic modalities to treat pancreatic cancer are ever expanding and include surgery, radiotherapy, chemotherapy and now immunotherapy. To obtain clinically effective and meaningful antitumor responses, the successful execution of several interventions will be required. Preclinical studies suggest that immunotherapy combinations targeting distinct steps of antitumor immunity might be synergistic, resulting in stronger and more sustained responses that accomplish durable tumor destruction. Targeting all parts of immune activation, depletion of immunosuppressor cells, enhancing Ag release and presentation and activation of adaptive immunity is crucial to efficient cancer immunotherapy. Bacterial formulations like IMM-101, which do not follow a ‘classic’ approach, offer the benefits of a multitude of immune modulation pathways. This diversity of responses may carry the key for tumor control and overcoming resistance to treatments. Indeed, this approach demonstrates the importance of combining immunotherapy with chemotherapy in the metastatic pancreatic cancer setting, where smaller metastatic lesions lacking the dense desmoplastic stroma of the primary tumor may be more amenable to treatment. Controlling metastatic disease will be the key to achieve better survival outcomes for patients with pancreatic cancer.
